# EXPLORING INEQUALITIES IN HEALTHY AGING: MANY APPROACHES BUT WHICH IS MOST USEFUL FOR POLICY?

**DOI:** 10.1093/geroni/igad104.1792

**Published:** 2023-12-21

**Authors:** Barbara Hanratty, Graciela Muniz-Terrera

## Abstract

Promoting healthy ageing requires an in-depth understanding of health inequalities. There are many different ways in which experiences of health and care may be inequitable, and numerous ways of studying this. In this symposium, we present findings from five disparate studies, united by an intention to shed light on unequal ageing. Our work utilises individual characteristics (frailty), aspects of care received (polypharmacy), access to services (digitalisation), different data sources (social care) and evidence synthesis methods, to characterise inequalities in later life, and inform policy. We start with a multi-method study of digitalisation in health services during the coronavirus pandemic, examining the impact of digital exclusion on inequalities in later life. Analysis of cohort data from the English Longitudinal Study of Ageing investigates associations between socio-economic measures and frailty free life expectancy in the over 50s. The impact of years of education on polypharmacy is explored in the Cognitive Function and Ageing Studies, and the value of information on social care provision exploited in analysis of real-world data. The final presentation will cover the development of a conceptual framework to ensure inequalities are considered within evidence synthesis Discussion will draw together the commonalities and differences of these approaches, and consider which have utility for policy makers, and how methods and approaches can be combined to produce an in-depth understanding of inequalities in healthy ageing.

